# Metabolic Networks Influencing Skeletal Muscle Fiber Composition

**DOI:** 10.3389/fcell.2018.00125

**Published:** 2018-09-28

**Authors:** Isabelle Bourdeau Julien, Chantelle F. Sephton, Paul A. Dutchak

**Affiliations:** ^1^Department of Psychiatry and Neuroscience, Université Laval, Quebec, QC, Canada; ^2^CERVO Brain Research Centre, Quebec, QC, Canada

**Keywords:** mTORC1, GATOR1, nuclear hormone receptor (NHR), metabolism, warbug effect, muscle physiology

## Abstract

Advancements in metabolomic and genomic research tools are revealing new insights into how metabolic networks can influence skeletal muscle fiber composition. In this mini-review, we summarize the recent progress of metabolite-dependent signaling pathways and transcriptional regulators that control glycolytic and oxidative metabolism and ultimately influence the type of fibers in muscle depots. These mechanisms expand the role of metabolites beyond that of basic building blocks of cellular components, and illustrate how particular metabolites can take an active role in regulating metabolic homeostasis and fiber adaptation. As new metabolite-dependent mechanisms emerge, ongoing metabolomic studies have begun to help explain why distinct metabolic pathways are used in different biological contexts and widen the view of seminal observations like the Warburg effect.

## Introduction

The metabolic properties of skeletal muscle fibers are intimately related to the biological function of the tissues they comprise. Investigation of the mechanisms that control fiber composition in skeletal muscle is an active area of research, partially due to the intriguing ability of fibers to adapt to nutritional or physiological challenges at a molecular and phenotypic level (Green et al., 1992; Adams et al., 1993; Widrick et al., 2002; Eshima et al., 2017). The adaptive process is associated with changes in intracellular metabolism, gene expression, and contractility of the fibers, which can broadly affect the health of an individual. The contribution of specific metabolites to the regulation of fiber composition has largely remained unknown due to the integrative nature of metabolism in complex physiological systems. Recent studies, combining genetic and metabolomic approaches, have begun to reveal novel relationships between the metabolite-regulated pathways that can influence muscle fiber composition and the ability to undergo metabolic switching from oxidative phosphorylation to aerobic glycolysis, a process known as the Warburg effect in cancer cells. In this mini-review, we discuss the metabolic properties of skeletal muscle fiber types and highlight metabolite-dependent pathways that can influence fiber composition.

## Metabolic properties of muscle fibers

Skeletal muscle depots are composed of heterogeneous populations of muscle fibers that permit a broad range of functions. Extensive research has helped define distinct types of muscle fibers that are categorized as slow-twitch (type I) and fast-twitch (type II), which contribute to long-term endurance or powerful bursts of movement, respectively (Szent-Györgyi, 2004). Slow-twitch fibers are dense in mitochondria to allow high oxidative capacity and sustain long-term energy demands; whereas fast-twitch fibers are subdivided into fast-oxidative (type IIa) or fast-glycolytic (type IIb/x), which correlate with their mitochondrial density. While the quantity of mitochondria is distinct between these fiber types, studies have also shown differences in the metabolism and structural characteristics of the mitochondria between these fiber types (Anderson and Neufer, 2006; Picard et al., 2008; Mishra et al., 2015). The energy requirements of muscle fibers often correlate with the expression of major myosin heavy chain (MHC) isoforms, which determine the rate of cross-bridge cycling with type I being the slowest, type IIa intermediate, and IIX/b the fastest. The MHC isoforms are encoded by *Myh7, Myh2, Myh1*, and *Myh4*, which are expressed in type I, IIa, IIx, and IIb fibers, respectively (Schiaffino and Reggiani, 2011).

There is a tight regulation of glycolytic and oxidative pathways in muscle fibers to ensure ATP production meets the demand of the tissue. This is necessary because ATP turnover rates can increase over 100-fold in active muscle compared to rest (Gaitanos et al., 1993). When there is an immediate energy demand (i.e., sprinting), a rapid increase of glycolytic metabolism occurs and pyruvate is converted to lactate, reminiscent of the Warburg effect (Warburg et al., 1927). The importance of switching from oxidative phosphorylation to aerobic glycolysis, which is less efficient in generating ATP, is to increase metabolites like NAD+ that are needed to accommodate continued glycolysis and other aspects of cellular metabolism and growth. It is in this manner that skeletal muscle metabolism can recapitulate metabolic hallmarks observed in cancer cells (Lunt and Vander Heiden, 2011). Intriguingly, regulatory pathways that control the metabolic flexibility of skeletal muscle fibers are frequently associated with cancer cell metabolism, including the mTORC1 pathway that stimulates aerobic glycolysis when activated in muscle. Overtime, changes in the metabolic environment within different fiber types can activate cell signaling and transcriptional mechanisms that stimulate an adaptive process that causes phenotypic changes of the fibers, a process called fiber type switching.

## Metabolite-dependent signaling mechanisms regulating fiber composition

**Figure 1:**
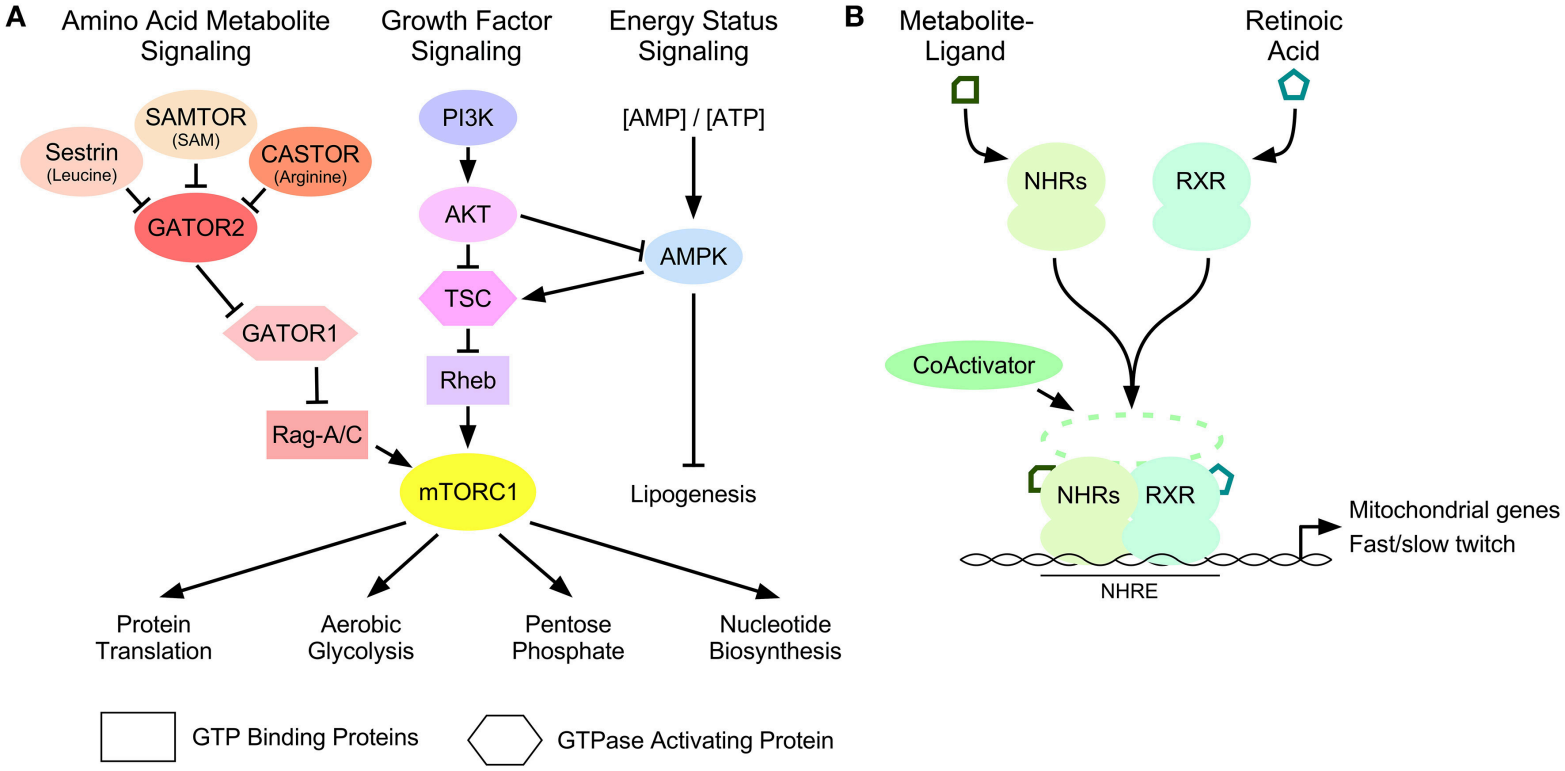
Metabolite-dependent mechanisms that influence muscle fiber composition. **(A)** The mTORC1 signal transduction pathway is controlled by amino acid-, growth factor- and energy-dependent signaling mechanisms. Amino acid-dependent signaling through Sestrin, CASTOR, and SAMTOR, repress GATOR2-dependent inhibition of GATOR1 GAP activity toward Rag GTP-binding proteins. Growth factor signaling through the phosphatidylinositol 3 kinase (PI3K): AKT pathway inhibits the GAP activity of the tuberous sclerosis complex (TSC) toward Rheb. These pathways lead to the stimulation of mTORC1 activity. Cellular energy status controls mTORC1 through a regulatory loop between the growth factor signaling pathway and the sensor of AMP called AMPK. Downstream targets of mTORC1 control protein translation and metabolic pathways that provide the substrates required for growth. **(B)** Ligand activation of nuclear hormone receptors (NHRs), including PPARδ and TRα, and form a heterodimer with retinoic acid receptor (RXR) that recruits coactivator proteins to the nuclear hormone response elements (NHRE) of the promoters of genes involved in mitochondrial gene expression and skeletal muscle fiber-type switching.

Cell signaling pathways control the homeostatic and adaptive properties of skeletal muscle fibers (Egan and Zierath, 2013). Several of these pathways are regulated by secondary messengers like cyclic adenosine monophosphate (Berdeaux and Stewart, 2012) or Ca^+2^ (Chin et al., 1998), whereas others are dependent on intracellular metabolites. Important metabolite-dependent pathways that effect skeletal muscle fiber composition and cellular metabolism in accordance with nutrient availability include the mammalian Target of Rapamycin (mTORC1) and AMP-activated protein kinase (AMPK) pathways (Fryer et al., 2002; Jørgensen et al., 2006; Philp et al., 2011) (Figure 1A). Cross-talk signaling between these pathways, beyond the scope of this review, antagonistically control the size of muscle cells (Mounier et al., 2011).

mTORC1 is a protein kinase that controls cellular metabolism and growth, in part, by stimulatory effects on protein translation (Düvel et al., 2010; Saxton and Sabatini, 2017). While the regulation of mTORC1 signaling is dependent on cell type and proliferative state (Laplante and Sabatini, 2012), it is also effected by metabolic stress in muscle fibers (Goodman et al., 2012). mTORC1 is activated by recruitment to the lysosomal surface where it interacts with small GTP-binding proteins called Rags or RHEB, which control mTORC1 activity as a function of amino acid availability or growth factor signaling, respectively (Figure 1A). The GTP-bound state of RHEB is controlled by the phosphatidylinositol 3-kinase/AKT signaling pathway, which inhibits the guanosine triphosphatase-activating protein (GAP) function of TSC1/2 toward RHEB, to permit mTORC1 activation (Inoki et al., 2002; Tee et al., 2002). In contrast, intracellular amino acids can regulate the GTP binding state of the Rag proteins by affecting the GAP activity of GATOR1. GATOR1 is an evolutionarily conserved complex comprised of three requisite proteins called nitrogen permease regulator-like 2 (NPRL2), nitrogen permease regulator-like 3 (NPRL3) and DEP domain containing protein 5 (DEPDC5) (Dokudovskaya et al., 2011; Wu and Tu, 2011). Low concentrations of intracellular amino acids cause GATOR1 dissociation from its negative regulatory complex called GATOR2, permitting GATOR1 to catalyze GTP-Rag hydrolysis to GDP-Rag and impair mTORC1 activity (Figure 1A) (Bar-Peled et al., 2013). The ability of GATOR2 to repress GATOR1 function is controlled by other proteins that respond to particular amino acids or their derivatives, including: Sestrin (leucine) (Parmigiani et al., 2014), CASTOR (arginine) (Chantranupong et al., 2016), and SAMTOR (S-adenosyl methionine) (Gu et al., 2017). The *in vivo* contribution of these upstream regulators of GATOR1 and their impact on skeletal muscle biology remains to be examined.

While each component of GATOR1 is necessary for embryonic development (Kowalczyk et al., 2012; Dutchak et al., 2015; Hughes et al., 2017), our recent studies show that loss of NPRL2 in skeletal muscle causes constitutive activation of mTORC1, aerobic glycolysis, and increased fast-twitch (type II) fibers in soleus muscle (Dutchak et al., 2018). Our observations, and others, indicate that mTORC1 regulates mitochondrial metabolism and controls mitochondrial-dependent synthesis of aspartate and glutamine for the generation of nitrogen-containing metabolites required for growth, while stimulating aerobic glycolysis to meet the cellular demands of ATP (Laxman et al., 2014; Birsoy et al., 2015; Chen et al., 2017; Dutchak et al., 2018). Importantly, the amino acids that activate mTORC1 can function as anaplerotic substrates in the mitochondria, consistent with these metabolites having an active role in regulating cellular homeostasis. During growth and proliferative stages, oxaloacetate, and α-ketoglutarate are converted to aspartate and glutamine in order to promote protein and nucleotide biosynthesis, rather than being used for oxidative metabolism (Dibble and Manning, 2013). If they are consumed for biosynthesis, they are no longer available for the generation of ATP by the mitochondria, and so the cells must upregulate glycolysis, leading to the Warburg effect. The selective nature of GATOR1 to respond to particular amino acids and subsequently influence carbohydrate and amino acid metabolism in the mitochondria highlights a fundamental and conserved aspect of metabolic homeostasis. It will be of interest for future studies to examine the contribution of individual amino acids toward regulating the glycolytic and oxidative pathways in different phases of skeletal muscle development.

## Metabolite-dependent transcriptional regulators of fiber composition

Long-term changes in skeletal muscle fiber composition are controlled by transcriptional mechanisms that regulate particular genetic programs important to each fiber type (Braun and Gautel, 2011). Early studies helped define signal transduction cascades as the major regulatory mechanism of transcription factors that contributes to fiber composition, like the calcineurin-dependent regulation of nuclear factor of activated T cells (NFAT) and myocyte enhancer factor 2 (MEF2) transcription factors, involved in the fast-to-slow twitch fiber transformation initiated by muscle contraction (Sreter et al., 1987; Kubis et al., 1997; Chin et al., 1998; Anderson et al., 2015). More recently, nuclear hormone receptor transcription factors have been shown to influence the type of fibers expressed in skeletal muscle. This class of transcription factors provide a direct link between intracellular metabolites and genomic expression as their ability to regulate gene transcription is dependent on ligand-activation by small molecules, or metabolites (Figure 1B) (Evans and Mangelsdorf, 2014).

A well categorized group of nuclear hormone receptors known as peroxisome-proliferating activated receptors (PPARs) are able to control cellular differentiation and metabolism when bound to their lipid-ligands. In skeletal muscle, PPARδ regulates genes important for fatty acid transport and oxidation, increasing lipid catabolism for energy production (Tanaka et al., 2003). Transgenic expression of lipid-activated PPARδ in skeletal muscle results in “marathon mice,” with increased slow-twitch oxidative muscle fibers, decreased fast-twitch fibers, and resistance to weight gain when fed high-fat diets that cause normal mice to become obese (Wang et al., 2004). Further transcriptional studies using pharmacological ligands to activate PPARδ showed it can induce the expression of its transcriptional co-activator called peroxisome proliferator activated-receptor-gamma co-activator-1 (PGC-1α) in muscle, which regulates mitochondrial gene expression (Lin et al., 2005; Hondares et al., 2007).

PGC1α is a co-activator of nuclear hormone receptors that can drive the formation of oxidative fiber-types and regulate the expression of specific genes important for oxidative metabolism (Lin et al., 2002; Olesen et al., 2010; Fernandez-Marcos and Auwerx, 2011). Overexpression of PGC-1α, using the muscle creatine kinase promoter, cause type II fibers to exhibit characteristics of type I fibers, with more myoglobin, troponin I (slow) and resistance to electrically stimulated fatigue (Lin et al., 2002). In an opposite manner, PGC-1α skeletal muscle knockout mice show a shift from type I and type Ia, to type IIx and IIb fibers (Handschin et al., 2007). PGC-1α activation is controlled by post-translational modification, including: (1) cell signaling networks, including AMPK, p38, PKA, AKT, (2) acetylation/deacetylation by GCN5 and SIRT1, respectively, and (3) methylation by PRMT1 (Fernandez-Marcos and Auwerx, 2011). Recently, studies of PGC-1α have identified multiple splice isoforms of the gene, and shown that novel variant called PGC-1α4 is increased with strength training (Ruas et al., 2012; Ydfors et al., 2013; Chan and Arany, 2014). Isoform PGC-1α4 regulates targets involved in two major signaling pathways, IGF1 signaling and myostatin, to promote strength and size of skeletal muscle (Ruas et al., 2012), in contrast to PGC-1α isoform 1 that promotes oxidative fibers (Lin et al., 2002).

The thyroid hormone nuclear receptor is activated by triiodothyronine (T3), a product of tyrosine metabolism, and contributes to skeletal muscle energy metabolism by regulating the transcription of mitochondrial genes and stimulating fiber type switching (Brent, 2012; De Andrade et al., 2015). T3 regulates the transcription of both nuclear and mitochondrial genes by binding to nuclear thyroid hormone receptors (TRα and TRβ) or a truncated forms of TRα called p43, located in the mitochondrial matrix (Brent, 2012; Lombardi et al., 2015). In mitochondrial matrix, T3 binding to p43 promotes transcription of mitochondrial genes involved in slow-twitch fiber metabolism, whereas p43 depletion has been shown to induce a switch to fast-twitch fibers and cause muscle hypertrophy (Pessemesse et al., 2011).

These studies highlight the importance of ligand-activated transcription factors and their co-activators in regulating mitochondrial biogenesis and fiber composition in muscle depots. Future approaches to refine the complex transcriptional networks involved in skeletal muscles physiology will benefit by using tissue specific models, as above, because confounding metabolic phenotypes can occur with whole body-knockout and transgenic expression systems.

## Future perspectives

The metabolic contribution to skeletal muscle fiber type composition is an important consideration for human health and disease. By combining scientific observations from exercise physiology to biochemistry, we are beginning to understand the logical basis of the intertwined nature of metabolism and skeletal muscle physiology. Important breakthroughs in metabolite-dependent cell signaling and transcriptional programs have highlighted new functions for metabolites in the muscle, well beyond that of the basic building blocks of cellular components. The mechanisms that drive these metabolite-dependent pathways to regulate short-term changes in glycolytic and oxidative metabolism in accordance with longer-term changes in fiber type composition will be important for future investigation. The phenotypic differences in skeletal muscle fiber composition that are caused by genetic alterations of these metabolite-dependent regulatory pathways, detailed above, suggest that different fiber type phenotypes ultimately emerge from differences in these metabolic effector networks. It will also be of interest to characterize the initial differences in metabolic-regulatory pathways in fiber types before switching occurs, to help determine why certain fiber types and muscle depots are more responsive to adaptation. How cells cope with transient fluctuations in metabolites is an important consideration that must be viewed in the context of the proliferation and growth of the cells.

Future progress to elucidate the fiber type specific mechanisms that regulate energy metabolism and control growth will help our understanding of how heterogeneous tissues can respond to their ever changing environment. In particular, metabolic mechanisms that regulate specific muscle fiber types will have important consequences for understanding human health and metabolism-related muscular atrophies, including cancer cachexia, sepsis, and diabetes. New discoveries in muscle may also further our understanding of oncogenic events, aging and neurodegenerative diseases like amyotrophic lateral sclerosis, Huntington's disease and Alzheimer's disease.

## Author contributions

All authors listed have made a substantial, direct and intellectual contribution to the work, and approved it for publication.

### Conflict of interest statement

The authors declare that the research was conducted in the absence of any commercial or financial relationships that could be construed as a potential conflict of interest.
